# 

**Published:** 1958-07

**Authors:** 


### *Contents*

| | | Page |
|---|---|---|
| EDITORIAL | *C.B.P.* | 55 |
| HOSPITAL CROSS-INFECTION | *Dr. W. A. Gillespie* | 56 |
| ON GROWING OLD | *Dr. J. H. Sheldon* | 69 |
| MEMINGOCOCCAL SEPTICAEMIA WITH BILATERAL ADRENAL HAEMORRHAGE (WATERHOUSE-FRIDERICHSEN SYNDROME) | *Clinical Pathological Conference* | 78 |
| NOTES AND NEWS | | 82 |
| APPOINTMENTS | | 83 |

